# CircAI: a comprehensive database of CircRNA associated with A-to-I RNA editing

**DOI:** 10.1093/database/baaf075

**Published:** 2025-11-22

**Authors:** Yulan Wang, Lingxiao Zou, Jian Zhao, Jing Wu, Meng Zhang, Jingjing Liu, Quan Wang, Xuejiang Guo, Xiaofeng Song, Yixuan Wang

**Affiliations:** Department of Biomedical Engineering, Nanjing University of Aeronautics and Astronautics, Nanjing 211106, China; School of Medical Information, Wannan Medical College, Wuhu 241002, China; Department of Biomedical Engineering, Nanjing University of Aeronautics and Astronautics, Nanjing 211106, China; Department of Biomedical Engineering, Nanjing University of Aeronautics and Astronautics, Nanjing 211106, China; School of Biomedical Engineering and Informatics, Nanjing Medical University, Nanjing 211166, China; Department of Biomedical Engineering, Nanjing University of Aeronautics and Astronautics, Nanjing 211106, China; Department of Biomedical Engineering, Nanjing University of Aeronautics and Astronautics, Nanjing 211106, China; Department of Biomedical Engineering, Nanjing University of Aeronautics and Astronautics, Nanjing 211106, China; State Key Laboratory of Reproductive Medicine and Offspring Health, Department of Histology and Embryology, Nanjing Medical University, Nanjing, Jiangsu 211166, China; Department of Biomedical Engineering, Nanjing University of Aeronautics and Astronautics, Nanjing 211106, China; Department of Biomedical Engineering, Nanjing University of Aeronautics and Astronautics, Nanjing 211106, China

## Abstract

RNA editing is a prevalent posttranscriptional modification characterized by single-base alterations in RNA transcripts, leading to diverse functional consequences, such as codon changes, mRNA splicing modulation, and regulation of noncoding RNAs, including their binding sites. Although next-generation sequencing has identified over 2 million adenosine-to-inosine (A-to-I) RNA editing sites in mammalian transcriptomes, the functional significance of the majority of these sites, especially those in noncoding regions, remains poorly understood. To address this gap and provide a comprehensive resource for exploring the functional impact of RNA editing in circular RNAs (circRNAs), we conducted an in-depth analysis of A-to-I editing sites in circRNAs across eight species (*Homo sapiens, Mus musculus, Macaca mulatta, Gallus gallus, Rattus norvegicus, Oryctolagus cuniculus, Sus scrofa*, and *Danio rerio*). All gathered data have been integrated into CircAI (circRNA associated with A-to-I RNA editing), the first database to combine multispecies circRNA editing data with functional predictions. CircAI offers a user-friendly platform for exploring the functional impact of RNA editing on circRNAs, including predictions of coding potential, miRNA interactions, secondary structures, and RNA editing quantitative trait loci (edQTL). By providing detailed annotations and dynamic visualization tools, CircAI serves as a pivotal resource for advancing research on the functional roles of RNA editing in circRNAs and their implications in disease.

**Database URL**: http://reprod.njmu.edu.cn/cgi-bin/circai/

## Introduction

In recent years, circular RNAs (circRNAs) have emerged as a central focus in noncoding RNA research due to their abundance and conservation across eukaryotes. Advances in high-throughput sequencing and bioinformatic tools have greatly accelerated circRNA discovery [1, 2]. CircRNAs are associated with genetic mutations, including single nucleotide polymorphisms (SNPs), and play important roles in human diseases [3]. In addition, RNA editing—particularly adenosine-to-inosine (A-to-I) editing catalysed by Adenosine deaminase acting on RNA (ADAR)—can modify circRNA sequences and influence their functions [4–6]. As the most prevalent RNA modification in metazoans, A-to-I editing contributes to various diseases, including neurological disorders and cancers [7].

Given the significance of circRNA and RNA editing, researchers have established several databases dedicated to these fields, including circRNADb [8], circBase [9], circAtlas [10], REDIportal [11, 12], and RADAR [13]. However, these resources exhibit notable limitations. For instance, circBase and circRNADb primarily focus on human circRNAs with limited species coverage. Similarly, although REDIportal and RADAR provide extensive catalogues of RNA editing events, they neither annotate the functional impact of these events nor explore their interaction with circRNAs. And circAtlas does not integrate RNA editing data.. These gaps hinder the exploration of the functional consequences of RNA editing on circRNAs, particularly in nonhuman species and disease contexts. To address these limitations, we developed CircAI, a unified platform that bridges RNA editing and circRNA research. As the first database to integrate multi-species circRNA editing data with functional predictions, CircAI provides a comprehensive resource for exploring the impact of RNA editing on circRNA functionality.

**Figure 1.**
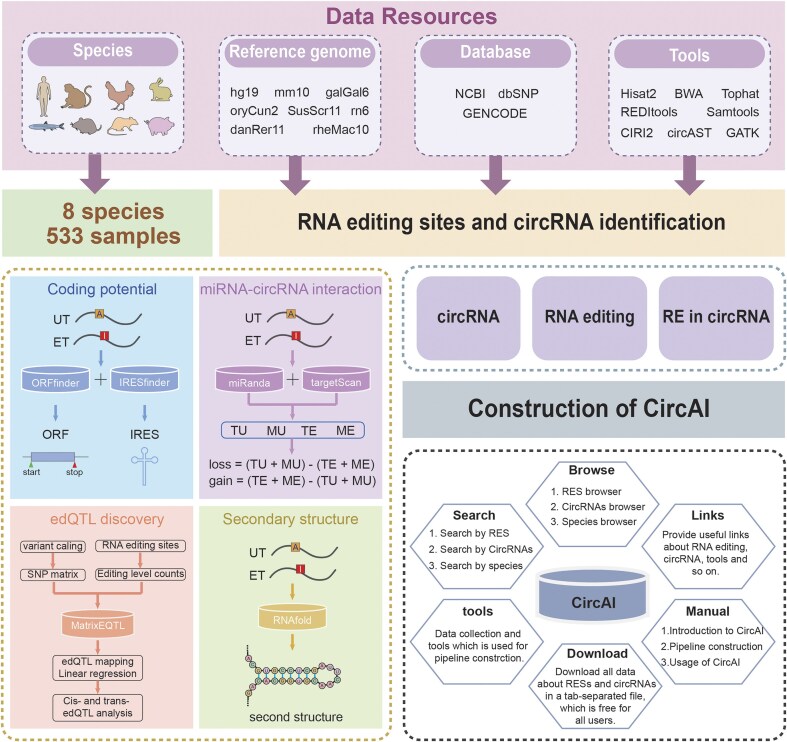
Workflow for the construction of CircAI.

CircAI focuses on three core objectives: (1) enabling cross-species comparison of circRNA editing events; (2) predicting the functional impact of editing on circRNA structure and interactions; and (3) identifying cancer-related editing sites to elucidate their roles in pathogenesis. Additionally, CircAI reconstructs full-length circRNA and provides detailed annotations including internal ribosomal entry sites (IRES), open reading frames (ORFs), RNA editing quantitative trait loci (edQTL), RNA secondary structures, and miRNA interactions (Fig. 1). Using dynamic visualization and efficient database management, CircAI offers user-friendly search, browse, and download capabilities, advancing the study of A-to-I editing in circRNAs. The CircAI database is freely accessible at http://reprod.njmu.edu.cn/cgi-bin/circai/.

## Materials and methods

### Database structure and functions

CircAI was constructed through comprehensive analysis of circRNAs and RNA editing events across 533 RNA-seq datasets from eight species (Fig. 1). CircRNAs were systematically identified using CIRI2 [14], and full-length sequences were reconstructed with CIRI Full and circAST pipelines. RNA editing sites within circRNA transcripts were detected by aligning genomic positions, and both edited type (ET) and unedited type (UT) sequences were generated for functional comparison. We assessed the impact of RNA editing on circRNA functionality through multiple approaches: coding potential was evaluated by predicting IRES and ORFs in both ET and UT sequences; circRNA–miRNA interactions were analysed using miRanda and TargetScan; RNA secondary structures were predicted with RNAfold; and edQTLs were identified for each species. Finally, CircAI provides a user-friendly web interface that supports search, browsing, visualization, and data download, facilitating exploration of circRNA editing events and their functional implications.

### Acquisition of RNA-seq samples

**Figure 2.**
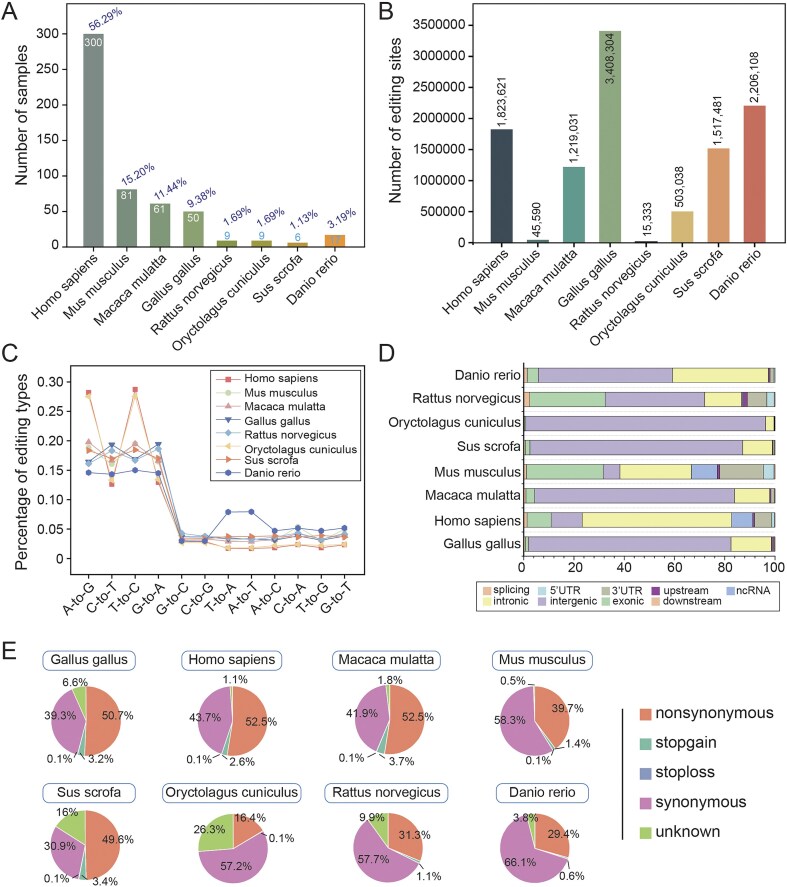
Global characteristics of RNA editing. (A) Number of samples for each species. (B) Number of editing sites identified in each species. (C) Number of 12 editing types per species. (D) Distribution of RNA editing sites in different genomic regions. (E) Functional annotations of RNA editing sites for each species.

To construct a high-confidence circRNA editing database, we systematically queried the NCBI SRA database using the search terms ‘RNA-seq’ and ‘RNase R’ to obtain RNase R-treated datasets. From these, we curated 533 RNA-seq samples spanning multiple species (Fig. 2A; Table S1). A standardized reference genome version (Table S2) was employed, with species-specific reference genome files, GTF annotation files, and RepeatMasker repeat annotation files sourced from the UCSC Genome Browser (http://genome.ucsc.edu). SNP data was obtained from dbSNP V151 (https://www.ncbi.nlm.nih.gov/snp).

### Detection and annotation of A-to-I RNA editing sites

To ensure the reliability of identification, REDITools [15] were utilized for the detection of A-to-I editing sites. Reads were first aligned to reference genome with HISAT2 [16], then subjected to stringent filtering: a minimum of 3 supported reads, at least 10 reads of total coverage, and an editing level ≥0.1. All known SNPs were excluded to minimize false positives. This comprehensive filtering approach yielded high-confidence, species-specific RNA editing sites (Fig. 2B), encompassing twelve editing categories across all species (Fig. 2C). Genomic annotationwas performed using ANNOVAR [17] with GENCODE gene models and UCSC RepeatMasker annotations, characterizing sites into nine genomic regions (Fig. 2D) and five functional categories based on amino acid change potential (Fig. 2E).

### CircRNA detection and full-length construction

**Figure 3.**
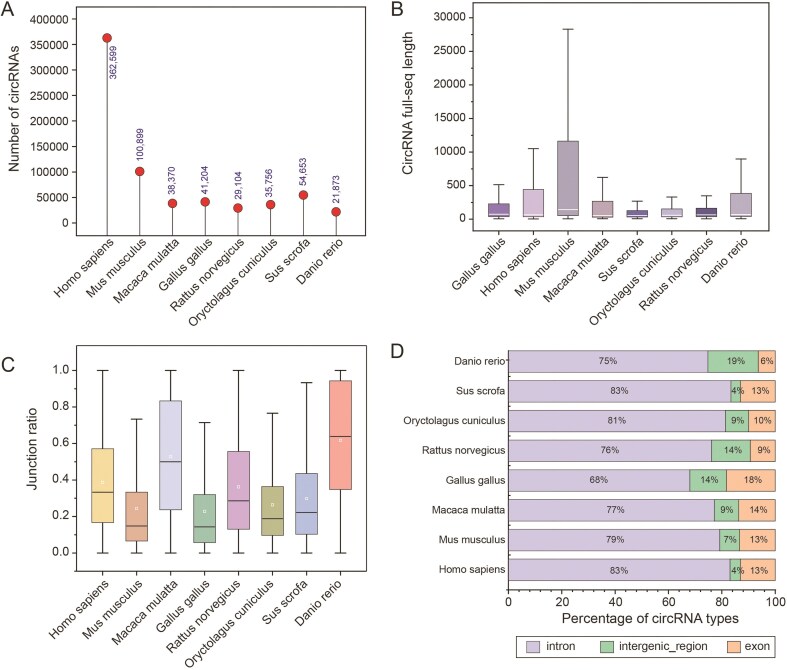
Characterization of different species of circRNAs. (A) Number of circRNAs in different species. (B) Full-length sequences of circRNAs for each species. (C) The junction ratio of circRNAs for each species. (D) Percentage of circRNA types.

For each RNA-seq dataset, sequencing reads were aligned to the reference genome using BWA MEM [18]. CircRNAs were identified using CIRI2 [14], retaining only those supported by at least two back-splice junction reads. Full-length circRNA sequences were reconstructed using CircAST [19] and an adjusted circFullSeq script, yielding high-confidence full-length circRNA sequences for each species. The results showed that the length distribution of circrnas in the eight species presented significant differences (Fig. 3A). For circRNAs not assembled by CircAST, a reference-based method was applied to connect exons and flanking intronic regions within the back-splice site (Fig. 3B). We further investigated the species specificity of circRNA biogenesis efficiency and observed pronounced variations among the eight species (Fig. 3C), suggesting that the biosynthesis efficiency or stability of circRNAs may be influenced by species-specific factors. Most circRNAs were classified as intronic types, followed by exonic and intergenic types (Fig. 3D).

### RNA editing in circRNA

**Figure 4:**
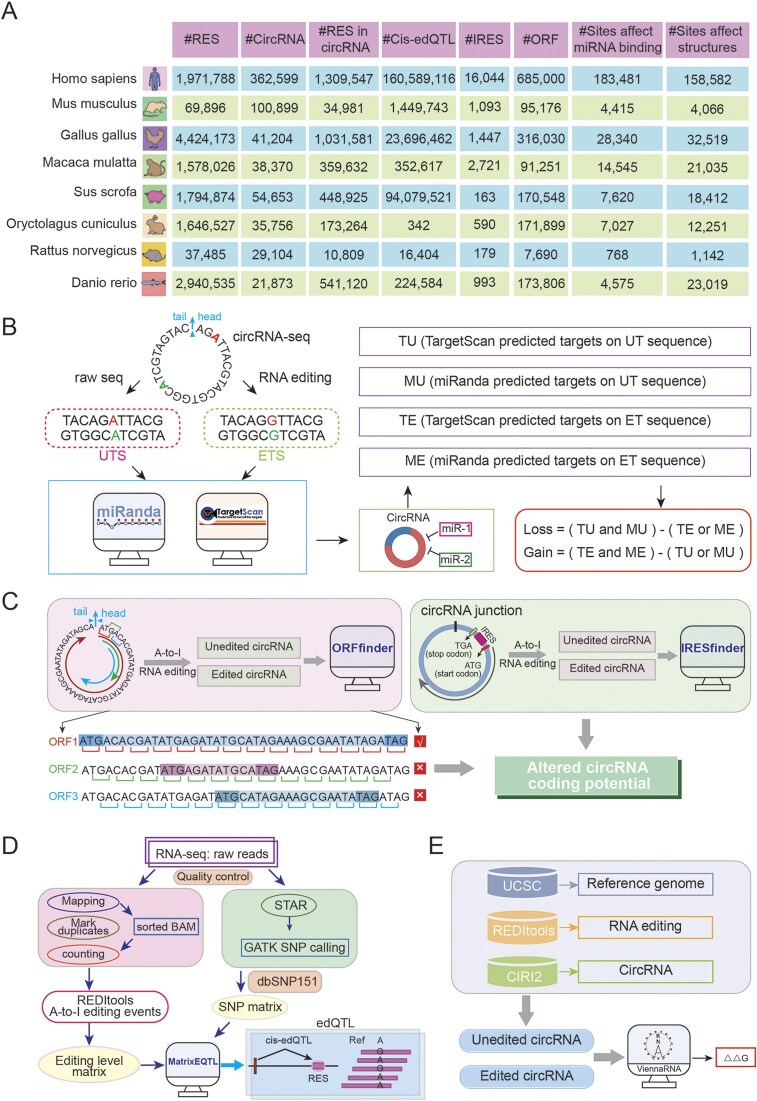
. CircAI database functional analysis framework. (A) Statistics of multispecies circRNA editing events. (B) Regulatory impact of RNA editing on circRNA–miRNA interactions. (C) RNA editing-mediated alterations in circRNA coding potential. (D) Workflow for identifying circRNA edQTLs. (E) Structural consequence analysis of RNA editing on circRNA secondary structure.

After standardizing reference genome and intersecting genomic locations, we acquired RNA editing sites within circRNAs for each species (Fig. 4A). These data were used to systematically evaluate the effects of RNA editing events on circRNA secondary structure, miRNA–circRNA interactions, and coding potential.

### Impacts of RNA editing sites on miRNA–circRNA interactions

Given that circRNAs can act as ‘sponges’ for miRNA, modulating gene expression and contributing to disease pathogenesis. We investigated how RNA editing influences miRNA–circRNA interactions. miRNA annotations were retrieved from miRBase. For each editing site, 51-bp sequences (25 bp flanking each side) were extracted from circRNA transcripts to generate UT sequences. The A nucleotide at the editing site was replaced with G to derive the ET sequence. TargetScan [20] and miRanda [21] were employed to predict miRNA targets on both UT and ET sequences. Four distinct target datasets were generated: TU, MU, TE, and ME. An interaction is categorized as ‘loss’ [Loss = (TU and MU)—(TE or ME)] when present in both UT and MT but not in both ET and ME. Conversely, an interaction is classified as ‘gain’ [Gain = (TE and ME)—(TU or MU)] if present in both ET and ME but not in both UT and MU (Fig. 4B). This comprehensive analysis revealed numerous editing sites that potentially disrupt existing or create new miRNA target sites.

### RNA editing affects circRNA protein coding potential

Although circRNAs lack of 5′ and 3′ endshas and are traditionally classified as noncoding RNAs, emerging evidence suggests they may possess protein-coding potential through internal IRES elements. Notably, the presence of start codon and ORFs in many circRNAs further supports this possibility. To assess whether RNA editing influences the coding capacity of circRNAs, we investigated the impact of RNA editing events on the coding capacity of circRNA in each species. The longest ORF was identified by concatenating three copies of each circRNA sequence to simulate circular continuity, then scanning all reading frames to select the maximum-length ORF (Fig. 4C). IRESfinder [22] was used to predict coding score for both UT and ET sequences, enabling comparison of coding potential alterations resulting from RNA editing (Fig. 4C).

### Identification of edQTLs

edQTL analysis links RNA editing levels with genotypes and has been widely utilized in genetic studies. Only SNPs within 200 kb of editing site were considered to focus on cis-regulatory effects. Associations between genotypes and editing frequencies were assessed using matrixEQTL [23] (Fig. 4D), with the most significant proximal SNP designated as the lead edQTL for each site.

### Impact of RNA editing on circRNA secondary structure

To evaluate structural consequences of editing, we predicted local RNA structures using a 51-bp window (±25 nt) centred on each editing site. The RNAfold [24] tool computed minimum free energy and optimal structures for both UT and ET sequences, enabling quantification of RNA stability changes induced by editing (Fig. 4E).

## Results

### Web implementation and usage instructions

**Figure 5.**
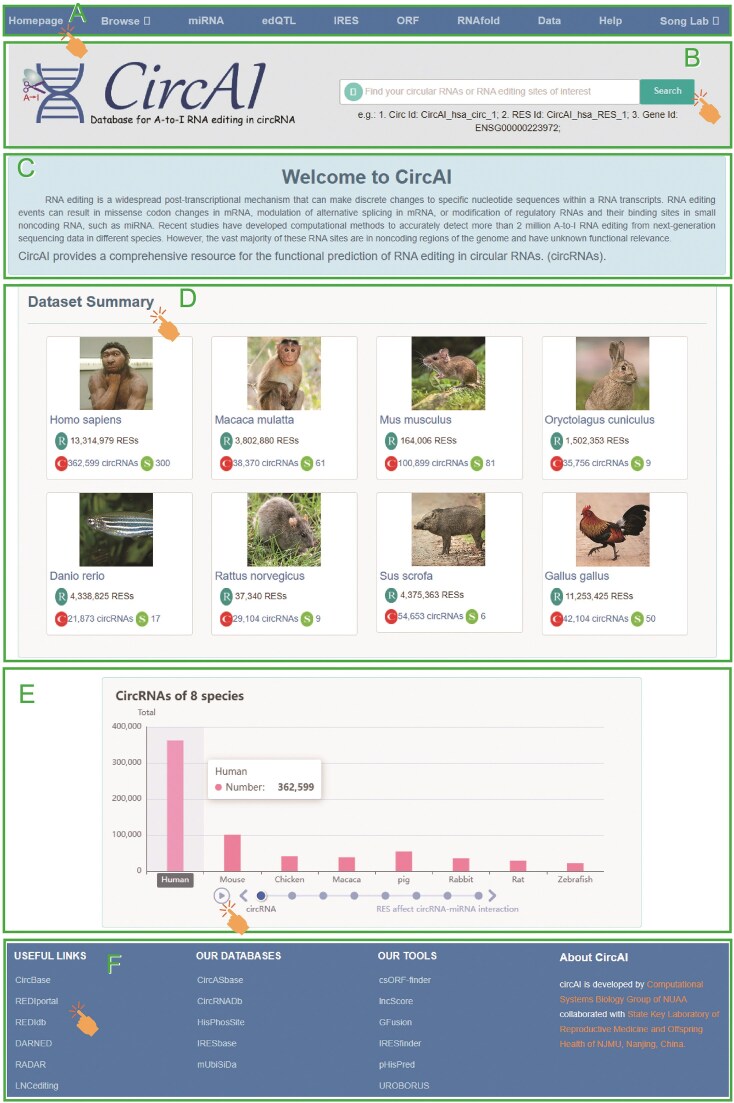
Comprehensive view of CircAI database.

CircAI offers a user-friendly, open-access web interface for efficient exploration, retrieval, and downloading of circRNA editing data and the corresponding functional annotations (Fig. 5). The homepage offers direct access to all database sections via a navigation bar, allowing users to browse data by species or functional module.

The homepage provides centralized navigation through a main menu, allowing direct access to species-specific or module-based exploration of the database. Users can quickly return to the homepage from any section and utilize a versatile search function to query by circRNA, editing site, gene identifier, or other relevant keywords. The browsing interface features organized dropdown menus for detailed molecular information under ‘All circRNA’ and a filterable display of RNA editing sites under ‘All RES’. Dedicated pages provide access to functional annotations including miRNA interactions, edQTL associations, IRES elements, ORF predictions, and RNA secondary structures. All data are available for download in the ‘Data’ section, while the ‘Help’ page offers detailed documentation describing data sources, analytical methods, and usage guidelines.

**Figure 6.**
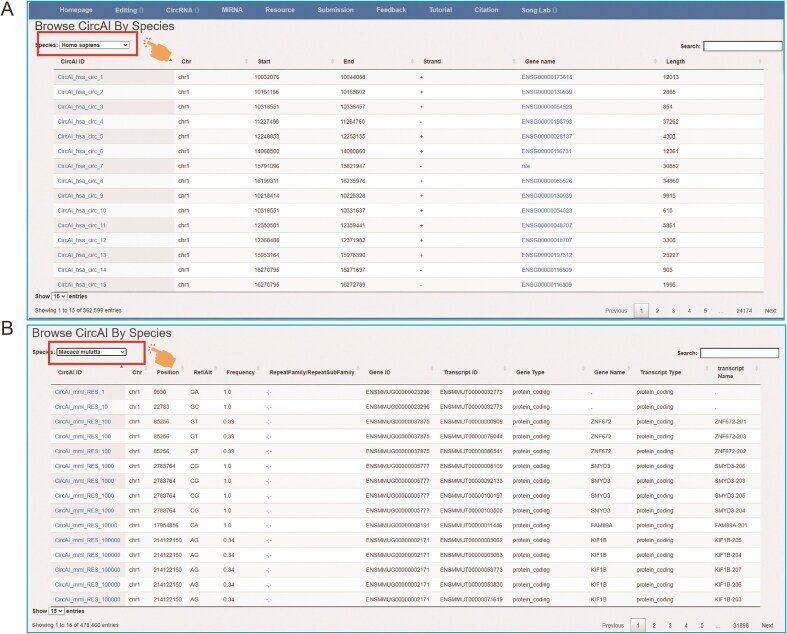
The page of CircAI’s (A) circRNA and (B) RNA editing detailed information.

To enhance database usability, CircAI incorporates dynamic visualization of RNA editing information, with results presented in interactive tables that support sorting and filtering (Fig. 6). The platform also provides links to related external databases and tools, as well as resources developed by our team, further supporting research in circRNA and RNA editing.

### Database organization and web interface

**Figure 7.**
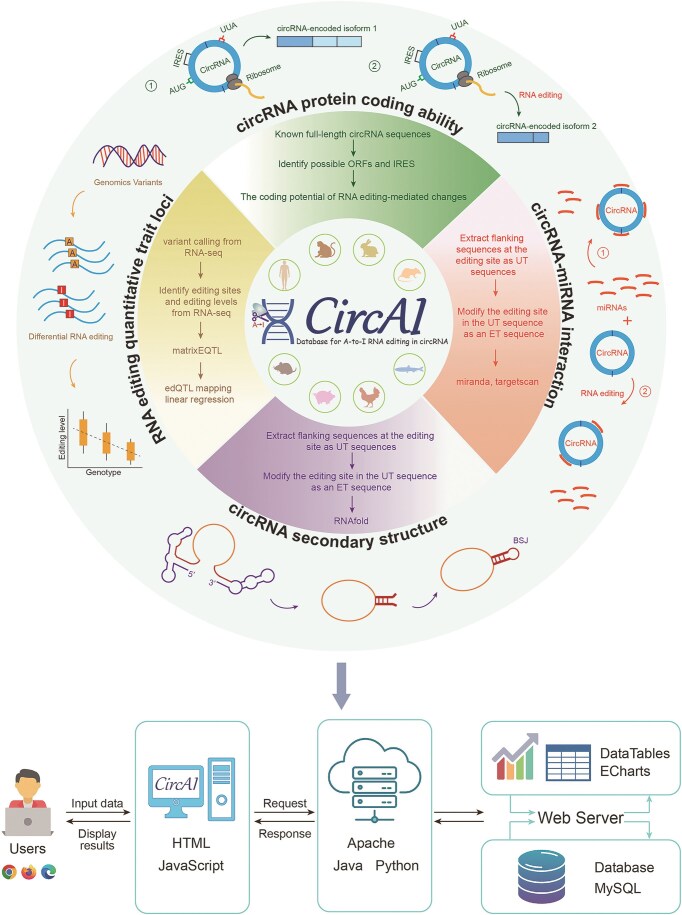
Architecture design of CircAI.

CircAI, as a novel platform for RNA editing and circRNA research, was constructed by assembling RNA editing sites, circRNA full sequence, and structural information as well as functional annotations. It was configured in a typical XAMPP (XWindows/Linux/Mac OS + Apache + MySQL + PHP + Perl) integrated environment (Fig. 7).

The user interface of CircAI is designed and implemented using JavaScript to enhance user-friendliness and operational experience. All data tables in CircAI are based on the jQuery table plugin DataTables, enabling clear and concise data display as well as providing filtering, sorting, and search functions for convenient data browsing and analysis. The web service of CircAI has been tested on popular browsers such as Google Chrome, Edge, and Firefox.

## Discussion and perspectives

CircAI is multispecies database dedicated to A-to-I RNA editing and its functional impact on circRNAs. It integrates comprehensive information on RNA editing, circRNA, and editing-mediated circRNA modifications, with visualization of how individual base changes affect coding potential, RBPs binding and miRNA interactions. By prioritizing critical circRNAs through these annotations, CircAI supports the systematic evaluation of RNA editing-mediated regulatory mechanisms. Encompassing samples from healthy tissues of eight species, CircAI presents the most comprehensive circRNA editing expression atlas currently available. This resource provides novel insights into the structural and functional alterations induced by RNA editing across species, significantly advancing our understanding of circRNA biology.

CircAI surpasses previous databases in the following aspects: (1) it compiles data from healthy tissues of seven species besides humans, presenting the most extensive circRNA editing atlas to date, fostering advancements in circRNA research and providing a collaborative data repository for researchers; (2) it incorporates detailed functional annotation of circRNA regulatory networks influenced by RNA editing alterations, encompassing miRNA and RBP interactions, coding potential, and corresponding genetic variations, enhancing researchers’ comprehension of RNA editing’s impact on circRNAs’ functions; (3) through the examination of the relationship between editing events in circRNAs and cancer, CircAI can offer valuable insights into cancer pathogenesis.

In the subsequent work, CircAI can be further strengthened in the following aspects: (1) incorporation of single-cell RNA-seq data to enable resolution at the cellular level, despite technical challenges related to circRNA capture efficiency in poly(A)-selected libraries [25, 26]; (2) incorporation of data related to drug targets. Chemoresistance poses a significant challenge in treating various cancers, with growing evidence linking circRNAs and RNA editing events to this phenomenon [27–30]. CircRNAs exhibiting RNA editing modifications may present novel therapeutic targets and prognostic biomarkers for cancer. Therefore, the inclusion of such data is essential for exploring the therapeutic potential of circRNAs; and (3) expansion to include additional species. This expansion is particularly crucial in the context of plant species, where RNA editing is prevalent, aiming to transform CircAI into a comprehensive database spanning diverse organisms, including animals and plants.

In conclusion, CircAI, a database focused on circRNA editing, serves as a valuable resource for investigating A-to-I RNA editing in noncoding RNA, and also provides the necessary data and platform for research on diseases and cancer.

## Supplementary Material

baaf075_Supplemental_File

## Data Availability

CircAI is a data resource portal that is freely available at http://reprod.njmu.edu.cn/cgi-bin/circai/.
