# Supplementary material for: CircAI: a comprehensive database of CircRNA associated with A-to-I RNA editing
Source: Database (Oxford). 2025 Nov 22;2025:baaf075. doi: 10.1093/database/baaf075 (PMC12639330; doi:10.1093/database/baaf075)
Supplement: baaf075_Supplemental_File [file baaf075_supplemental_file.docx]

**CircAI: A Comprehensive Database of CircRNA Associated With A-to-I RNA Editing**

Yulan Wang^1,2^, Lingxiao Zou^1^, Jian Zhao^1^, Jing Wu^2^, Meng Zhang^1^, Jingjing Liu^1^, Quan Wang^1^, Xuejiang Guo^3,^*, Xiaofeng Song^1,^* and Yixuan Wang^1,^*

^1^ Department of Biomedical Engineering, Nanjing University of Aeronautics and Astronautics, Nanjing 211106, China

^2^ School of Medical Information, Wannan Medical College, Wuhu 241002, China.

^3^ School of Biomedical Engineering and Informatics, Nanjing Medical University, Nanjing 211166, China

^4^ State Key Laboratory of Reproductive Medicine and Offspring Health, Department of Histology and Embryology, Nanjing Medical University, Nanjing, Jiangsu 211166, China

* Corresponding authors.

Email: [guo_xuejiang@njmu.edu.cn](mailto:guo_xuejiang@njmu.edu.cn) (Guo X), [xfsong@nuaa.edu.cn](mailto:xfsong@nuaa.edu.cn) (Song X), [wangyixuan@nuaa.edu.cn](mailto:wangyixuan@nuaa.edu.cn) (Wang Y).

**Supplementary Tables**

**Table S1.** Summary of sample information for the in-house and public cohorts used in this study**.**

**Table S2.** UCSC genome version and annotation files for each species used in the CircAI database.

**Supplementary Tables**

**Table S1.** Summary of sample information in the CircAI database.

| **Species** | **SRA_Accession** | **PMID** |
| --- | --- | --- |
| Gallus gallus | SRR10247291 | 31948317 |
| Gallus gallus | SRR10247290 | 31948317 |
| Gallus gallus | SRR10247289 | 31948317 |
| Gallus gallus | SRR10247288 | 31948317 |
| Gallus gallus | SRR10247287 | 31948317 |
| Gallus gallus | SRR10247286 | 31948317 |
| Gallus gallus | SRR10247285 | 31948317 |
| Gallus gallus | SRR10247284 | 31948317 |
| Gallus gallus | SRR10247283 | 31948317 |
| Gallus gallus | SRR10247282 | 31948317 |
| Gallus gallus | SRR10247281 | 31948317 |
| Gallus gallus | SRR10247280 | 31948317 |
| Gallus gallus | SRR10247279 | 31948317 |
| Gallus gallus | SRR10247278 | 31948317 |
| Gallus gallus | SRR7524136 | 30700247 |
| Gallus gallus | SRR7524137 | 30700247 |
| Gallus gallus | SRR7524138 | 30700247 |
| Gallus gallus | SRR7524139 | 30700247 |
| Gallus gallus | SRR7524140 | 30700247 |
| Gallus gallus | SRR7524141 | 30700247 |
| Gallus gallus | SRR7524142 | 30700247 |
| Gallus gallus | SRR7524143 | 30700247 |
| Gallus gallus | SRR7524144 | 30700247 |
| Gallus gallus | SRR8369114 | 32365656 |
| Gallus gallus | SRR8369115 | 32365656 |
| Gallus gallus | SRR8369116 | 32365656 |
| Gallus gallus | SRR8369117 | 32365656 |
| Gallus gallus | SRR8369118 | 32365656 |
| Gallus gallus | SRR8369119 | 32365656 |
| Gallus gallus | SRR8369120 | 32365656 |
| Gallus gallus | SRR8369121 | 32365656 |
| Gallus gallus | SRR8369122 | 32365656 |
| Gallus gallus | SRR7700649 | 31214170 |
| Gallus gallus | SRR7700650 | 31214170 |
| Gallus gallus | SRR7700651 | 31214170 |
| Gallus gallus | SRR7700652 | 31214170 |
| Gallus gallus | SRR7700653 | 31214170 |
| Gallus gallus | SRR7700654 | 31214170 |
| Gallus gallus | SRR4734702 | 29036326 |
| Gallus gallus | SRR4734703 | 29036326 |
| Gallus gallus | SRR4734704 | 29036326 |
| Gallus gallus | SRR4734705 | 29036326 |
| Gallus gallus | SRR4734706 | 29036326 |
| Gallus gallus | SRR4734707 | 29036326 |
| Gallus gallus | SRR5599677 | 30608205 |
| Gallus gallus | SRR5599678 | 30608205 |
| Gallus gallus | SRR5599679 | 30608205 |
| Gallus gallus | SRR5599680 | 30608205 |
| Gallus gallus | SRR5599681 | 30608205 |
| Gallus gallus | SRR5599682 | 30608205 |
| Homo sapiens | SRR10357392 | 32223537 |
| Homo sapiens | SRR10357391 | 32223537 |
| Homo sapiens | SRR10357390 | 32223537 |
| Homo sapiens | SRR10357389 | 32223537 |
| Homo sapiens | SRR10357388 | 32223537 |
| Homo sapiens | SRR10357387 | 32223537 |
| Homo sapiens | SRR10357386 | 32223537 |
| Homo sapiens | SRR10357385 | 32223537 |
| Homo sapiens | SRR10357384 | 32223537 |
| Homo sapiens | SRR8713146 | 33325513 |
| Homo sapiens | SRR8713147 | 33325513 |
| Homo sapiens | SRR8713148 | 33325513 |
| Homo sapiens | SRR8713149 | 33325513 |
| Homo sapiens | SRR8713150 | 33325513 |
| Homo sapiens | SRR8713151 | 33325513 |
| Homo sapiens | SRR8713153 | 33325513 |
| Homo sapiens | SRR8713154 | 33325513 |
| Homo sapiens | SRR8713155 | 33325513 |
| Homo sapiens | SRR8713156 | 33325513 |
| Homo sapiens | SRR8713157 | 33325513 |
| Homo sapiens | SRR8713158 | 33325513 |
| Homo sapiens | SRR8713159 | 33325513 |
| Homo sapiens | SRR8713160 | 33325513 |
| Homo sapiens | SRR8713161 | 33325513 |
| Homo sapiens | SRR8713166 | 33325513 |
| Homo sapiens | SRR8713167 | 33325513 |
| Homo sapiens | SRR8713181 | 33325513 |
| Homo sapiens | SRR8713182 | 33325513 |
| Homo sapiens | SRR8713197 | 33325513 |
| Homo sapiens | SRR8713198 | 33325513 |
| Homo sapiens | SRR8713199 | 33325513 |
| Homo sapiens | SRR8713200 | 33325513 |
| Homo sapiens | SRR8713206 | 33325513 |
| Homo sapiens | SRR8713207 | 33325513 |
| Homo sapiens | SRR8713208 | 33325513 |
| Homo sapiens | SRR8713209 | 33325513 |
| Homo sapiens | SRR8713210 | 33325513 |
| Homo sapiens | SRR8713211 | 33325513 |
| Homo sapiens | SRR8713212 | 33325513 |
| Homo sapiens | SRR8713213 | 33325513 |
| Homo sapiens | SRR8713214 | 33325513 |
| Homo sapiens | SRR8713215 | 33325513 |
| Homo sapiens | SRR9080661 | 31616472 |
| Homo sapiens | SRR9080660 | 31616472 |
| Homo sapiens | SRR9080659 | 31616472 |
| Homo sapiens | SRR9080658 | 31616472 |
| Homo sapiens | SRR9080657 | 31616472 |
| Homo sapiens | SRR9080656 | 31616472 |
| Homo sapiens | SRR8375640 | 30849979 |
| Homo sapiens | SRR8375639 | 30849979 |
| Homo sapiens | SRR8375638 | 30849979 |
| Homo sapiens | SRR8375637 | 30849979 |
| Homo sapiens | SRR8375636 | 30849979 |
| Homo sapiens | SRR8375635 | 30849979 |
| Homo sapiens | SRR7611489 | 30150410 |
| Homo sapiens | SRR7611490 | 30150410 |
| Homo sapiens | SRR7611491 | 30150410 |
| Homo sapiens | SRR7611492 | 30150410 |
| Homo sapiens | SRR7611493 | 30150410 |
| Homo sapiens | SRR7611494 | 30150410 |
| Homo sapiens | SRR7611495 | 30150410 |
| Homo sapiens | SRR7611496 | 30150410 |
| Homo sapiens | SRR7611497 | 30150410 |
| Homo sapiens | SRR7611498 | 30150410 |
| Homo sapiens | SRR6807289 | 30365065 |
| Homo sapiens | SRR6807290 | 30365065 |
| Homo sapiens | SRR6807291 | 30365065 |
| Homo sapiens | SRR6807292 | 30365065 |
| Homo sapiens | SRR6807293 | 30365065 |
| Homo sapiens | SRR6807294 | 30365065 |
| Homo sapiens | SRR5991126 | 30497055 |
| Homo sapiens | SRR5991127 | 30497055 |
| Homo sapiens | SRR5991134 | 30497055 |
| Homo sapiens | SRR5991135 | 30497055 |
| Homo sapiens | SRR5991136 | 30497055 |
| Homo sapiens | SRR5991147 | 30497055 |
| Homo sapiens | SRR6328687 | 29415187 |
| Homo sapiens | SRR6328688 | 29415187 |
| Homo sapiens | SRR6328689 | 29415187 |
| Homo sapiens | SRR6328690 | 29415187 |
| Homo sapiens | SRR6328691 | 29415187 |
| Homo sapiens | SRR6328692 | 29415187 |
| Homo sapiens | SRR6328693 | 29415187 |
| Homo sapiens | SRR6328694 | 29415187 |
| Homo sapiens | SRR6328695 | 29415187 |
| Homo sapiens | SRR6328696 | 29415187 |
| Homo sapiens | SRR17235470 | 36304262 |
| Homo sapiens | SRR17235469 | 36304262 |
| Homo sapiens | SRR17235468 | 36304262 |
| Homo sapiens | SRR17235467 | 36304262 |
| Homo sapiens | SRR17235466 | 36304262 |
| Homo sapiens | SRR17235465 | 36304262 |
| Homo sapiens | [SRR15561384](https://trace.ncbi.nlm.nih.gov/Traces/sra/?run=SRR15561386) | 36304262 |
| Homo sapiens | SRR15561385 | 36304262 |
| Homo sapiens | SRR15561386 | 36304262 |
| Homo sapiens | [SRR15403023](https://trace.ncbi.nlm.nih.gov/Traces/sra/?run=SRR15403023) | 36732659 |
| Homo sapiens | SRR15403024 | 36732659 |
| Homo sapiens | SRR15403025 | 36732659 |
| Homo sapiens | SRR15403026 | 36732659 |
| Homo sapiens | SRR15403027 | 36732659 |
| Homo sapiens | SRR15403028 | 36732659 |
| Homo sapiens | SRR15403029 | 36732659 |
| Homo sapiens | SRR15403030 | 36732659 |
| Homo sapiens | SRR15403031 | 36732659 |
| Homo sapiens | SRR15403032 | 36732659 |
| [Homo sapiens](https://www.ncbi.nlm.nih.gov/Taxonomy/Browser/wwwtax.cgi?mode=Info&id=9606) | [SRR13961698](https://trace.ncbi.nlm.nih.gov/Traces/sra/?run=SRR13961698) | 34933019 |
| [Homo sapiens](https://www.ncbi.nlm.nih.gov/Taxonomy/Browser/wwwtax.cgi?mode=Info&id=9606) | [SRR13961699](https://trace.ncbi.nlm.nih.gov/Traces/sra/?run=SRR13961698) | 34933019 |
| Homo sapiens | SRR13194213 | 33323517 |
| Homo sapiens | SRR13194214 | 33323517 |
| Homo sapiens | SRR13194215 | 33323517 |
| [Homo sapiens](https://www.ncbi.nlm.nih.gov/Taxonomy/Browser/wwwtax.cgi?mode=Info&id=9606) | [SRR12781544](https://trace.ncbi.nlm.nih.gov/Traces/sra/?run=SRR12781544) | 34123587 |
| Homo sapiens | SRR12781545 | 34123587 |
| Homo sapiens | SRR12781546 | 34123587 |
| Homo sapiens | SRR12781547 | 34123587 |
| Homo sapiens | SRR12781548 | 34123587 |
| Homo sapiens | SRR12781549 | 34123587 |
| [Homo sapiens](https://www.ncbi.nlm.nih.gov/Taxonomy/Browser/wwwtax.cgi?mode=Info&id=9606) | [SRR11735816](https://trace.ncbi.nlm.nih.gov/Traces/sra/?run=SRR11735816) | 33230478 |
| Homo sapiens | SRR11735817 | 33230478 |
| Homo sapiens | SRR11735818 | 33230478 |
| Homo sapiens | SRR11735819 | 33230478 |
| Homo sapiens | SRR11735820 | 33230478 |
| Homo sapiens | SRR11735821 | 33230478 |
| Homo sapiens | [SRR11735815](https://trace.ncbi.nlm.nih.gov/Traces/sra/?run=SRR11735815) | 33230478 |
| Homo sapiens | [SRR11735813](https://trace.ncbi.nlm.nih.gov/Traces/sra/?run=SRR11735813) | 33230478 |
| Homo sapiens | [SRR11735814](https://trace.ncbi.nlm.nih.gov/Traces/sra/?run=SRR11735814) | 33230478 |
| Homo sapiens | [SRR11496508](https://trace.ncbi.nlm.nih.gov/Traces/sra/?run=SRR11496508) | 33363159 |
| Homo sapiens | SRR11496509 | 33363159 |
| Homo sapiens | SRR11496510 | 33363159 |
| Homo sapiens | SRR11496511 | 33363159 |
| [Homo sapiens](https://www.ncbi.nlm.nih.gov/Taxonomy/Browser/wwwtax.cgi?mode=Info&id=9606) | [SRR11178348](https://trace.ncbi.nlm.nih.gov/Traces/sra/?run=SRR11178348) | 32790025 |
| Homo sapiens | SRR11178349 | 32790025 |
| Homo sapiens | SRR11178350 | 32790025 |
| Homo sapiens | SRR11178351 | 32790025 |
| Homo sapiens | SRR11178352 | 32790025 |
| Homo sapiens | SRR11178353 | 32790025 |
| Homo sapiens | [SRR11095575](https://trace.ncbi.nlm.nih.gov/Traces/sra/?run=SRR11095575) | 32312268 |
| Homo sapiens | [SRR11095576](https://trace.ncbi.nlm.nih.gov/Traces/sra/?run=SRR11095575) | 32312268 |
| Homo sapiens | [SRR10612068](https://trace.ncbi.nlm.nih.gov/Traces/sra/?run=SRR10612068) | 33436621 |
| Homo sapiens | SRR10612069 | 33436621 |
| Homo sapiens | SRR10612070 | 33436621 |
| Homo sapiens | SRR10522343 | / |
| Homo sapiens | SRR10522342 | / |
| Homo sapiens | SRR10522341 | / |
| Homo sapiens | [SRR10522340](https://trace.ncbi.nlm.nih.gov/Traces/sra/?run=SRR10522340) | / |
| Homo sapiens | [SRR10277186](https://trace.ncbi.nlm.nih.gov/Traces/sra/?run=SRR10277186) | 32757146 |
| Homo sapiens | [SRR10277187](https://trace.ncbi.nlm.nih.gov/Traces/sra/?run=SRR10277186) | 32757146 |
| Homo sapiens | [SRR9855315](https://trace.ncbi.nlm.nih.gov/Traces/sra/?run=SRR9855315) | 31409897 |
| Homo sapiens | [SRR9855316](https://trace.ncbi.nlm.nih.gov/Traces/sra/?run=SRR9855315) | 31409897 |
| [Homo sapiens](https://www.ncbi.nlm.nih.gov/Taxonomy/Browser/wwwtax.cgi?mode=Info&id=9606) | [SRR9722032](https://trace.ncbi.nlm.nih.gov/Traces/sra/?run=SRR9722032) | 32719345 |
| [Homo sapiens](https://www.ncbi.nlm.nih.gov/Taxonomy/Browser/wwwtax.cgi?mode=Info&id=9606) | [SRR9722033](https://trace.ncbi.nlm.nih.gov/Traces/sra/?run=SRR9722032) | 32719345 |
| [Homo sapiens](https://www.ncbi.nlm.nih.gov/Taxonomy/Browser/wwwtax.cgi?mode=Info&id=9606) | [SRR9624078](https://trace.ncbi.nlm.nih.gov/Traces/sra/?run=SRR9624078) | 32232013 |
| Homo sapiens | SRR9624079 | 32232013 |
| Homo sapiens | SRR9624080 | 32232013 |
| Homo sapiens | SRR9624081 | 32232013 |
| Homo sapiens | SRR9624082 | 32232013 |
| Homo sapiens | SRR9624083 | 32232013 |
| [Homo sapiens](https://www.ncbi.nlm.nih.gov/Taxonomy/Browser/wwwtax.cgi?mode=Info&id=9606) | [SRR7350933](https://trace.ncbi.nlm.nih.gov/Traces/sra/?run=SRR7350933) | 30660194 |
| [Homo sapiens](https://www.ncbi.nlm.nih.gov/Taxonomy/Browser/wwwtax.cgi?mode=Info&id=9606) | [SRR8182800](https://trace.ncbi.nlm.nih.gov/Traces/sra/?run=SRR8182800) | 30537115 |
| Homo sapiens | SRR8182801 | 30537115 |
| Homo sapiens | SRR8182802 | 30537115 |
| Homo sapiens | SRR8182803 | 30537115 |
| Homo sapiens | SRR8182804 | 30537115 |
| Homo sapiens | SRR8182805 | 30537115 |
| Homo sapiens | SRR8182806 | 30537115 |
| Homo sapiens | SRR8182807 | 30537115 |
| Homo sapiens | SRR8182808 | 30537115 |
| Homo sapiens | SRR8182809 | 30537115 |
| Homo sapiens | SRR8182810 | 30537115 |
| Homo sapiens | SRR8182811 | 30537115 |
| Homo sapiens | SRR8182812 | 30537115 |
| Homo sapiens | SRR8182813 | 30537115 |
| Homo sapiens | SRR8182814 | 30537115 |
| Homo sapiens | SRR8182815 | 30537115 |
| Homo sapiens | SRR8182816 | 30537115 |
| Homo sapiens | SRR8182817 | 30537115 |
| Homo sapiens | SRR8182818 | 30537115 |
| Homo sapiens | SRR8182819 | 30537115 |
| [Homo sapiens](https://www.ncbi.nlm.nih.gov/Taxonomy/Browser/wwwtax.cgi?mode=Info&id=9606) | [SRR8107700](https://trace.ncbi.nlm.nih.gov/Traces/sra/?run=SRR8107700) | 30455306 |
| Homo sapiens | SRR8107701 | 30455306 |
| Homo sapiens | SRR8107702 | 30455306 |
| Homo sapiens | SRR8107703 | 30455306 |
| Homo sapiens | SRR8107704 | 30455306 |
| Homo sapiens | SRR8107705 | 30455306 |
| Homo sapiens | SRR8107706 | 30455306 |
| Homo sapiens | SRR8107707 | 30455306 |
| Homo sapiens | [SRR8074317](https://trace.ncbi.nlm.nih.gov/Traces/sra/?run=SRR8074317) | 32164722 |
| Homo sapiens | SRR8074318 | 32164722 |
| Homo sapiens | SRR8074319 | 32164722 |
| Homo sapiens | SRR8074320 | 32164722 |
| Homo sapiens | SRR8074321 | 32164722 |
| Homo sapiens | SRR8074322 | 32164722 |
| Homo sapiens | SRR8074323 | 32164722 |
| Homo sapiens | SRR8074324 | 32164722 |
| [Homo sapiens](https://www.ncbi.nlm.nih.gov/Taxonomy/Browser/wwwtax.cgi?mode=Info&id=9606) | [SRR7798700](https://trace.ncbi.nlm.nih.gov/Traces/sra/?run=SRR7798700) | 30455306 |
| Homo sapiens | SRR7798701 | 30455306 |
| Homo sapiens | SRR7798702 | 30455306 |
| Homo sapiens | SRR7798703 | 30455306 |
| Homo sapiens | SRR7798704 | 30455306 |
| Homo sapiens | SRR7798705 | 30455306 |
| Homo sapiens | SRR7798706 | 30455306 |
| Homo sapiens | SRR7798707 | 30455306 |
| Homo sapiens | SRR7798708 | 30455306 |
| Homo sapiens | SRR7798709 | 30455306 |
| Homo sapiens | SRR7798710 | 30455306 |
| Homo sapiens | SRR7798711 | 30455306 |
| Homo sapiens | [SRR7798712](https://trace.ncbi.nlm.nih.gov/Traces/sra/?run=SRR15561386) | 30455306 |
| Homo sapiens | [SRR7798713](https://trace.ncbi.nlm.nih.gov/Traces/sra/?run=SRR15561386) | 30455306 |
| Homo sapiens | SRR7798714 | 30455306 |
| Homo sapiens | [SRR7798715](https://trace.ncbi.nlm.nih.gov/Traces/sra/?run=SRR15403023) | 30455306 |
| Homo sapiens | [SRR7798716](https://trace.ncbi.nlm.nih.gov/Traces/sra/?run=SRR15403023) | 30455306 |
| Homo sapiens | [SRR7798717](https://trace.ncbi.nlm.nih.gov/Traces/sra/?run=SRR15403023) | 30455306 |
| Homo sapiens | [SRR7798718](https://trace.ncbi.nlm.nih.gov/Traces/sra/?run=SRR15403023) | 30455306 |
| Homo sapiens | [SRR7798719](https://trace.ncbi.nlm.nih.gov/Traces/sra/?run=SRR15403023) | 30455306 |
| [Homo sapiens](https://www.ncbi.nlm.nih.gov/Taxonomy/Browser/wwwtax.cgi?mode=Info&id=9606) | [SRR7474065](https://trace.ncbi.nlm.nih.gov/Traces/sra/?run=SRR7474066) | 30080890 |
| Homo sapiens | [SRR7474066](https://trace.ncbi.nlm.nih.gov/Traces/sra/?run=SRR7474066) | 30080890 |
| Homo sapiens | [SRR7474067](https://trace.ncbi.nlm.nih.gov/Traces/sra/?run=SRR15403023) | 30080890 |
| Homo sapiens | [SRR7474068](https://trace.ncbi.nlm.nih.gov/Traces/sra/?run=SRR15403023) | 30080890 |
| Homo sapiens | SRR7474069 | 30080890 |
| Homo sapiens | SRR7474070 | 30080890 |
| Homo sapiens | SRR7474071 | 30080890 |
| Homo sapiens | [SRR7474072](https://trace.ncbi.nlm.nih.gov/Traces/sra/?run=SRR13311620) | 30080890 |
| Homo sapiens | [SRR7474073](https://trace.ncbi.nlm.nih.gov/Traces/sra/?run=SRR13311620) | 30080890 |
| Homo sapiens | [SRR7474074](https://trace.ncbi.nlm.nih.gov/Traces/sra/?run=SRR13311620) | 30080890 |
| Homo sapiens | [SRR7474075](https://trace.ncbi.nlm.nih.gov/Traces/sra/?run=SRR13311620) | 30080890 |
| Homo sapiens | [SRR7474076](https://trace.ncbi.nlm.nih.gov/Traces/sra/?run=SRR13311620) | 30080890 |
| Homo sapiens | SRR7474077 | 30080890 |
| Homo sapiens | SRR7474078 | 30080890 |
| Homo sapiens | SRR7474079 | 30080890 |
| Homo sapiens | [SRR6999001](https://trace.ncbi.nlm.nih.gov/Traces/sra/?run=SRR6999001) | 30735634 |
| Homo sapiens | SRR6999002 | 30735634 |
| [Homo sapiens](https://www.ncbi.nlm.nih.gov/Taxonomy/Browser/wwwtax.cgi?mode=Info&id=10090) | [SRR6999003](https://trace.ncbi.nlm.nih.gov/Traces/sra/?run=SRR12809340) | 30735634 |
| [Homo sapiens](https://www.ncbi.nlm.nih.gov/Taxonomy/Browser/wwwtax.cgi?mode=Info&id=10090) | [SRR6999004](https://trace.ncbi.nlm.nih.gov/Traces/sra/?run=SRR12809340) | 30735634 |
| [Homo sapiens](https://www.ncbi.nlm.nih.gov/Taxonomy/Browser/wwwtax.cgi?mode=Info&id=10090) | [SRR6999005](https://trace.ncbi.nlm.nih.gov/Traces/sra/?run=SRR12809340) | 30735634 |
| [Homo sapiens](https://www.ncbi.nlm.nih.gov/Taxonomy/Browser/wwwtax.cgi?mode=Info&id=10090) | [SRR6999006](https://trace.ncbi.nlm.nih.gov/Traces/sra/?run=SRR12809340) | 30735634 |
| [Homo sapiens](https://www.ncbi.nlm.nih.gov/Taxonomy/Browser/wwwtax.cgi?mode=Info&id=10090) | [SRR6999007](https://trace.ncbi.nlm.nih.gov/Traces/sra/?run=SRR12809340) | 30735634 |
| Homo sapiens | SRR6999008 | 30735634 |
| Homo sapiens | SRR6999009 | 30735634 |
| Homo sapiens | SRR6999010 | 30735634 |
| Homo sapiens | SRR6999011 | 30735634 |
| Homo sapiens | SRR6999012 | 30735634 |
| Homo sapiens | SRR6999013 | 30735634 |
| Homo sapiens | SRR6999014 | 30735634 |
| Homo sapiens | SRR6999015 | 30735634 |
| Homo sapiens | SRR6999016 | 30735634 |
| Homo sapiens | SRR6999017 | 30735634 |
| Homo sapiens | SRR6999018 | 30735634 |
| Homo sapiens | SRR6999019 | 30735634 |
| Homo sapiens | SRR6999020 | 30735634 |
| Homo sapiens | SRR6999021 | 30735634 |
| Homo sapiens | SRR6999022 | 30735634 |
| Homo sapiens | SRR6999023 | 30735634 |
| Homo sapiens | SRR6999024 | 30735634 |
| Homo sapiens | SRR6505105 | 32624692 |
| Homo sapiens | SRR6505104 | 32624692 |
| Homo sapiens | SRR6505103 | 32624692 |
| Homo sapiens | SRR6505102 | 32624692 |
| [Homo sapiens](https://www.ncbi.nlm.nih.gov/Taxonomy/Browser/wwwtax.cgi?mode=Info&id=9606) | [SRR6505101](https://trace.ncbi.nlm.nih.gov/Traces/sra/?run=SRR12781544) | 32624692 |
| [Homo sapiens](https://www.ncbi.nlm.nih.gov/Taxonomy/Browser/wwwtax.cgi?mode=Info&id=9606) | [SRR6505100](https://trace.ncbi.nlm.nih.gov/Traces/sra/?run=SRR12781544) | 32624692 |
| [Homo sapiens](https://www.ncbi.nlm.nih.gov/Taxonomy/Browser/wwwtax.cgi?mode=Info&id=9606) | [SRR5901119](https://trace.ncbi.nlm.nih.gov/Traces/sra/?run=SRR12781544) | 30176335 |
| [Homo sapiens](https://www.ncbi.nlm.nih.gov/Taxonomy/Browser/wwwtax.cgi?mode=Info&id=9606) | [SRR5901120](https://trace.ncbi.nlm.nih.gov/Traces/sra/?run=SRR12781544) | 30176335 |
| [Homo sapiens](https://www.ncbi.nlm.nih.gov/Taxonomy/Browser/wwwtax.cgi?mode=Info&id=9606) | [SRR5901121](https://trace.ncbi.nlm.nih.gov/Traces/sra/?run=SRR12781544) | 30176335 |
| Homo sapiens | SRR5901122 | 30176335 |
| [Homo sapiens](https://www.ncbi.nlm.nih.gov/Taxonomy/Browser/wwwtax.cgi?mode=Info&id=9606) | [SRR5901123](https://trace.ncbi.nlm.nih.gov/Traces/sra/?run=SRR11735816) | 30176335 |
| [Homo sapiens](https://www.ncbi.nlm.nih.gov/Taxonomy/Browser/wwwtax.cgi?mode=Info&id=9606) | [SRR5901124](https://trace.ncbi.nlm.nih.gov/Traces/sra/?run=SRR11735816) | 30176335 |
| [Homo sapiens](https://www.ncbi.nlm.nih.gov/Taxonomy/Browser/wwwtax.cgi?mode=Info&id=9606) | [SRR5398213](https://trace.ncbi.nlm.nih.gov/Traces/sra/?run=SRR5398213) | 28794202 |
| [Homo sapiens](https://www.ncbi.nlm.nih.gov/Taxonomy/Browser/wwwtax.cgi?mode=Info&id=9606) | [SRR5398214](https://trace.ncbi.nlm.nih.gov/Traces/sra/?run=SRR11735816) | 28794202 |
| [Homo sapiens](https://www.ncbi.nlm.nih.gov/Taxonomy/Browser/wwwtax.cgi?mode=Info&id=9606) | [SRR5398215](https://trace.ncbi.nlm.nih.gov/Traces/sra/?run=SRR11735816) | 28794202 |
| [Homo sapiens](https://www.ncbi.nlm.nih.gov/Taxonomy/Browser/wwwtax.cgi?mode=Info&id=9606) | SRR5398216 | 28794202 |
| [Homo sapiens](https://www.ncbi.nlm.nih.gov/Taxonomy/Browser/wwwtax.cgi?mode=Info&id=9606) | SRR5398217 | 28794202 |
| [Homo sapiens](https://www.ncbi.nlm.nih.gov/Taxonomy/Browser/wwwtax.cgi?mode=Info&id=9606) | SRR5398218 | 28794202 |
| [Homo sapiens](https://www.ncbi.nlm.nih.gov/Taxonomy/Browser/wwwtax.cgi?mode=Info&id=9606) | [SRR5122011](https://trace.ncbi.nlm.nih.gov/Traces/sra/?run=SRR5122011) | 28444238/ 31980816 |
| [Homo sapiens](https://www.ncbi.nlm.nih.gov/Taxonomy/Browser/wwwtax.cgi?mode=Info&id=9606) | [SRR5122012](https://trace.ncbi.nlm.nih.gov/Traces/sra/?run=SRR11496508) | 28444238/ 31980816 |
| [Homo sapiens](https://www.ncbi.nlm.nih.gov/Taxonomy/Browser/wwwtax.cgi?mode=Info&id=9606) | [SRR3479143](https://trace.ncbi.nlm.nih.gov/Traces/sra/?run=SRR3479143) | 27350239 |
| [Homo sapiens](https://www.ncbi.nlm.nih.gov/Taxonomy/Browser/wwwtax.cgi?mode=Info&id=9606) | [SRR3479144](https://trace.ncbi.nlm.nih.gov/Traces/sra/?run=SRR11496508) | 27350239 |
| Homo sapiens | SRR3479145 | 27350239 |
| [Homo sapiens](https://www.ncbi.nlm.nih.gov/Taxonomy/Browser/wwwtax.cgi?mode=Info&id=9823) | [SRR3479116](https://trace.ncbi.nlm.nih.gov/Traces/sra/?run=SRR3479116) | 27350239 |
| [Homo sapiens](https://www.ncbi.nlm.nih.gov/Taxonomy/Browser/wwwtax.cgi?mode=Info&id=9823) | [SRR1637089](https://trace.ncbi.nlm.nih.gov/Traces/sra/?run=SRR1637089) | 27350239 |
| [Homo sapiens](https://www.ncbi.nlm.nih.gov/Taxonomy/Browser/wwwtax.cgi?mode=Info&id=9823) | [SRR1637090](https://trace.ncbi.nlm.nih.gov/Traces/sra/?run=SRR1637089) | 27350239 |
| [Homo sapiens](https://www.ncbi.nlm.nih.gov/Taxonomy/Browser/wwwtax.cgi?mode=Info&id=9823) | [SRR3476958](https://trace.ncbi.nlm.nih.gov/Traces/sra/?run=SRR3476958) | 27350239 |
| [Homo sapiens](https://www.ncbi.nlm.nih.gov/Taxonomy/Browser/wwwtax.cgi?mode=Info&id=9823) | [SRR1636985](https://trace.ncbi.nlm.nih.gov/Traces/sra/?run=SRR1636985) | 27350239 |
| Homo sapiens | [SRR3476956](https://trace.ncbi.nlm.nih.gov/Traces/sra/?run=SRR3476956) | 27350239 |
| [Homo sapiens](https://www.ncbi.nlm.nih.gov/Taxonomy/Browser/wwwtax.cgi?mode=Info&id=9606) | [SRR2318063](https://trace.ncbi.nlm.nih.gov/Traces/sra/?run=SRR2318063) | 28611215 |
| [Homo sapiens](https://www.ncbi.nlm.nih.gov/Taxonomy/Browser/wwwtax.cgi?mode=Info&id=9606) | [SRR2318064](https://trace.ncbi.nlm.nih.gov/Traces/sra/?run=SRR11178348) | 28611215 |
| [Homo sapiens](https://www.ncbi.nlm.nih.gov/Taxonomy/Browser/wwwtax.cgi?mode=Info&id=9606) | [SRR2318065](https://trace.ncbi.nlm.nih.gov/Traces/sra/?run=SRR11178348) | 28611215 |
| [Homo sapiens](https://www.ncbi.nlm.nih.gov/Taxonomy/Browser/wwwtax.cgi?mode=Info&id=9606) | [SRR2318066](https://trace.ncbi.nlm.nih.gov/Traces/sra/?run=SRR11178348) | 28611215 |
| [Homo sapiens](https://www.ncbi.nlm.nih.gov/Taxonomy/Browser/wwwtax.cgi?mode=Info&id=9606) | [SRR2318067](https://trace.ncbi.nlm.nih.gov/Traces/sra/?run=SRR11178348) | 28611215 |
| [Homo sapiens](https://www.ncbi.nlm.nih.gov/Taxonomy/Browser/wwwtax.cgi?mode=Info&id=9606) | SRR2318068 | 28611215 |
| [Homo sapiens](https://www.ncbi.nlm.nih.gov/Taxonomy/Browser/wwwtax.cgi?mode=Info&id=9606) | [SRR1772957](https://trace.ncbi.nlm.nih.gov/Traces/sra/?run=SRR1772957) | / |
| [Homo sapiens](https://www.ncbi.nlm.nih.gov/Taxonomy/Browser/wwwtax.cgi?mode=Info&id=9606) | [SRR1772257](https://trace.ncbi.nlm.nih.gov/Traces/sra/?run=SRR1772257) | / |
| [Homo sapiens](https://www.ncbi.nlm.nih.gov/Taxonomy/Browser/wwwtax.cgi?mode=Info&id=9606) | [SRR1777309](https://trace.ncbi.nlm.nih.gov/Traces/sra/?run=SRR1777309) | / |
| [Homo sapiens](https://www.ncbi.nlm.nih.gov/Taxonomy/Browser/wwwtax.cgi?mode=Info&id=9606) | [SRR1777310](https://trace.ncbi.nlm.nih.gov/Traces/sra/?run=SRR1777310) | / |
| [Homo sapiens](https://www.ncbi.nlm.nih.gov/Taxonomy/Browser/wwwtax.cgi?mode=Info&id=9606) | [SRR1049826](https://trace.ncbi.nlm.nih.gov/Traces/sra/?run=SRR1049826) | 25561518 |
| [Homo sapiens](https://www.ncbi.nlm.nih.gov/Taxonomy/Browser/wwwtax.cgi?mode=Info&id=9606) | [SRR1049827](https://trace.ncbi.nlm.nih.gov/Traces/sra/?run=SRR10522340) | 25561518 |
| [Homo sapiens](https://www.ncbi.nlm.nih.gov/Taxonomy/Browser/wwwtax.cgi?mode=Info&id=9606) | [SRR1049828](https://trace.ncbi.nlm.nih.gov/Traces/sra/?run=SRR10522340) | 25561518 |
| [Homo sapiens](https://www.ncbi.nlm.nih.gov/Taxonomy/Browser/wwwtax.cgi?mode=Info&id=9606) | [SRR1049829](https://trace.ncbi.nlm.nih.gov/Traces/sra/?run=SRR10522340) | 25561518 |
| [Homo sapiens](https://www.ncbi.nlm.nih.gov/Taxonomy/Browser/wwwtax.cgi?mode=Info&id=9606) | SRR1049830 | 25561518 |
| [Homo sapiens](https://www.ncbi.nlm.nih.gov/Taxonomy/Browser/wwwtax.cgi?mode=Info&id=9606) | SRR1049831 | 25561518 |
| Homo sapiens | SRR1049832 | 25561518 |
| [Homo sapiens](https://www.ncbi.nlm.nih.gov/Taxonomy/Browser/wwwtax.cgi?mode=Info&id=10090) | [SRR1049833](https://trace.ncbi.nlm.nih.gov/Traces/sra/?run=SRR10004190) | 25561518 |
| [Homo sapiens](https://www.ncbi.nlm.nih.gov/Taxonomy/Browser/wwwtax.cgi?mode=Info&id=9606) | [SRR6505115](https://trace.ncbi.nlm.nih.gov/Traces/sra/?run=SRR6505115) | / |
| [Macaca mulatta](https://www.ncbi.nlm.nih.gov/Taxonomy/Browser/wwwtax.cgi?mode=Info&id=10090) | [SRR5198454](https://trace.ncbi.nlm.nih.gov/Traces/sra/?run=SRR10004190) | 30245844 |
| [Macaca mulatta](https://www.ncbi.nlm.nih.gov/Taxonomy/Browser/wwwtax.cgi?mode=Info&id=9606) | SRR5198455 | 30245844 |
| [Macaca mulatta](https://www.ncbi.nlm.nih.gov/Taxonomy/Browser/wwwtax.cgi?mode=Info&id=9606) | SRR5198456 | 30245844 |
| Macaca mulatta | SRR5198457 | 30245844 |
| Macaca mulatta | SRR5198458 | 30245844 |
| Macaca mulatta | SRR5198459 | 30245844 |
| [Macaca mulatta](https://www.ncbi.nlm.nih.gov/Taxonomy/Browser/wwwtax.cgi?mode=Info&id=9606) | [SRR5198460](https://trace.ncbi.nlm.nih.gov/Traces/sra/?run=SRR9624078) | 30245844 |
| [Macaca mulatta](https://www.ncbi.nlm.nih.gov/Taxonomy/Browser/wwwtax.cgi?mode=Info&id=9606) | [SRR5198461](https://trace.ncbi.nlm.nih.gov/Traces/sra/?run=SRR9624078) | 30245844 |
| [Macaca mulatta](https://www.ncbi.nlm.nih.gov/Taxonomy/Browser/wwwtax.cgi?mode=Info&id=9606) | [SRR5198462](https://trace.ncbi.nlm.nih.gov/Traces/sra/?run=SRR9624078) | 30245844 |
| [Macaca mulatta](https://www.ncbi.nlm.nih.gov/Taxonomy/Browser/wwwtax.cgi?mode=Info&id=9606) | [SRR5198463](https://trace.ncbi.nlm.nih.gov/Traces/sra/?run=SRR9624078) | 30245844 |
| [Macaca mulatta](https://www.ncbi.nlm.nih.gov/Taxonomy/Browser/wwwtax.cgi?mode=Info&id=9606) | [SRR5198464](https://trace.ncbi.nlm.nih.gov/Traces/sra/?run=SRR9624078) | 30245844 |
| Macaca mulatta | SRR5198465 | 30245844 |
| [Macaca mulatta](https://www.ncbi.nlm.nih.gov/Taxonomy/Browser/wwwtax.cgi?mode=Info&id=9544) | [SRR5198466](https://trace.ncbi.nlm.nih.gov/Traces/sra/?run=SRR8401385) | 30245844 |
| [Macaca mulatta](https://www.ncbi.nlm.nih.gov/Taxonomy/Browser/wwwtax.cgi?mode=Info&id=9544) | [SRR5198467](https://trace.ncbi.nlm.nih.gov/Traces/sra/?run=SRR8401385) | 30245844 |
| Macaca mulatta | SRR5198468 | 30245844 |
| Macaca mulatta | SRR5198469 | 30245844 |
| Macaca mulatta | SRR5198470 | 30245844 |
| Macaca mulatta | SRR5198471 | 30245844 |
| Macaca mulatta | SRR5198472 | 30245844 |
| Macaca mulatta | SRR5198473 | 30245844 |
| Macaca mulatta | SRR5198474 | 30245844 |
| Macaca mulatta | SRR5198443 | 30245844 |
| Macaca mulatta | SRR5198444 | 30245844 |
| Macaca mulatta | SRR5198445 | 30245844 |
| Macaca mulatta | SRR5198446 | 30245844 |
| Macaca mulatta | SRR5198447 | 30245844 |
| Macaca mulatta | SRR5198448 | 30245844 |
| Macaca mulatta | SRR5198449 | 30245844 |
| Macaca mulatta | SRR5198450 | 30245844 |
| Macaca mulatta | SRR5198451 | 30245844 |
| Macaca mulatta | SRR5198452 | 30245844 |
| Macaca mulatta | SRR5198453 | 30245844 |
| Macaca mulatta | SRR2337312 | 26546448 |
| Macaca mulatta | SRR2337313 | 26546448 |
| Macaca mulatta | SRR2337314 | 26546448 |
| Macaca mulatta | SRR2337315 | 26546448 |
| Macaca mulatta | SRR2337316 | 26546448 |
| [Macaca mulatta](https://www.ncbi.nlm.nih.gov/Taxonomy/Browser/wwwtax.cgi?mode=Info&id=9606) | [SRR2337317](https://trace.ncbi.nlm.nih.gov/Traces/sra/?run=SRR8107700) | 26546448 |
| [Macaca mulatta](https://www.ncbi.nlm.nih.gov/Taxonomy/Browser/wwwtax.cgi?mode=Info&id=9606) | [SRR2337318](https://trace.ncbi.nlm.nih.gov/Traces/sra/?run=SRR8107700) | 26546448 |
| [Macaca mulatta](https://www.ncbi.nlm.nih.gov/Taxonomy/Browser/wwwtax.cgi?mode=Info&id=9606) | [SRR2337319](https://trace.ncbi.nlm.nih.gov/Traces/sra/?run=SRR8107700) | 26546448 |
| [Macaca mulatta](https://www.ncbi.nlm.nih.gov/Taxonomy/Browser/wwwtax.cgi?mode=Info&id=9606) | [SRR2337320](https://trace.ncbi.nlm.nih.gov/Traces/sra/?run=SRR8107700) | 26546448 |
| [Macaca mulatta](https://www.ncbi.nlm.nih.gov/Taxonomy/Browser/wwwtax.cgi?mode=Info&id=9606) | [SRR2337321](https://trace.ncbi.nlm.nih.gov/Traces/sra/?run=SRR8107700) | 26546448 |
| [Macaca mulatta](https://www.ncbi.nlm.nih.gov/Taxonomy/Browser/wwwtax.cgi?mode=Info&id=9606) | [SRR2337322](https://trace.ncbi.nlm.nih.gov/Traces/sra/?run=SRR8107700) | 26546448 |
| [Macaca mulatta](https://www.ncbi.nlm.nih.gov/Taxonomy/Browser/wwwtax.cgi?mode=Info&id=9606) | [SRR2337323](https://trace.ncbi.nlm.nih.gov/Traces/sra/?run=SRR8107700) | 26546448 |
| [Macaca mulatta](https://www.ncbi.nlm.nih.gov/Taxonomy/Browser/wwwtax.cgi?mode=Info&id=9606) | SRR2337324 | 26546448 |
| [Macaca mulatta](https://www.ncbi.nlm.nih.gov/Taxonomy/Browser/wwwtax.cgi?mode=Info&id=9606) | [SRR2337325](https://trace.ncbi.nlm.nih.gov/Traces/sra/?run=SRR8074317) | 26546448 |
| [Macaca mulatta](https://www.ncbi.nlm.nih.gov/Taxonomy/Browser/wwwtax.cgi?mode=Info&id=9606) | [SRR2337326](https://trace.ncbi.nlm.nih.gov/Traces/sra/?run=SRR8074317) | 26546448 |
| [Macaca mulatta](https://www.ncbi.nlm.nih.gov/Taxonomy/Browser/wwwtax.cgi?mode=Info&id=9606) | [SRR2337327](https://trace.ncbi.nlm.nih.gov/Traces/sra/?run=SRR8074317) | 26546448 |
| [Macaca mulatta](https://www.ncbi.nlm.nih.gov/Taxonomy/Browser/wwwtax.cgi?mode=Info&id=9606) | [SRR2337328](https://trace.ncbi.nlm.nih.gov/Traces/sra/?run=SRR8074317) | 26546448 |
| [Macaca mulatta](https://www.ncbi.nlm.nih.gov/Taxonomy/Browser/wwwtax.cgi?mode=Info&id=9606) | [SRR2337329](https://trace.ncbi.nlm.nih.gov/Traces/sra/?run=SRR8074317) | 26546448 |
| [Macaca mulatta](https://www.ncbi.nlm.nih.gov/Taxonomy/Browser/wwwtax.cgi?mode=Info&id=9606) | [SRR2337330](https://trace.ncbi.nlm.nih.gov/Traces/sra/?run=SRR8074317) | 26546448 |
| [Macaca mulatta](https://www.ncbi.nlm.nih.gov/Taxonomy/Browser/wwwtax.cgi?mode=Info&id=9606) | [SRR2337331](https://trace.ncbi.nlm.nih.gov/Traces/sra/?run=SRR8074317) | 26546448 |
| Macaca mulatta | SRR2337332 | 26546448 |
| Macaca mulatta | SRR2337333 | 26546448 |
| Macaca mulatta | SRR2337334 | 26546448 |
| Macaca mulatta | SRR2337335 | 26546448 |
| Macaca mulatta | SRR2337336 | 26546448 |
| Macaca mulatta | SRR2337337 | 26546448 |
| [Macaca mulatta](https://www.ncbi.nlm.nih.gov/Taxonomy/Browser/wwwtax.cgi?mode=Info&id=9544) | [SRR8401385](https://trace.ncbi.nlm.nih.gov/Traces/sra/?run=SRR8401385) | 30567979 |
| Macaca mulatta | SRR8401386 | 30567979 |
| Macaca mulatta | SRR8401387 | 30567979 |
| Mus musculus | SRR9201206 | 31786335 |
| Mus musculus | SRR9201203 | 31786335 |
| Mus musculus | SRR9201200 | 31786335 |
| Mus musculus | SRR9201197 | 31786335 |
| Mus musculus | SRR9201194 | 31786335 |
| Mus musculus | SRR9201191 | 31786335 |
| Mus musculus | SRR9201188 | 31786335 |
| Mus musculus | SRR9201185 | 31786335 |
| Mus musculus | SRR9201182 | 31786335 |
| Mus musculus | SRR9201179 | 31786335 |
| Mus musculus | SRR9201176 | 31786335 |
| Mus musculus | SRR9201173 | 31786335 |
| Mus musculus | SRR8256801 | 30643264 |
| Mus musculus | SRR8256802 | 30643264 |
| [Mus musculus](https://www.ncbi.nlm.nih.gov/Taxonomy/Browser/wwwtax.cgi?mode=Info&id=9606) | SRR7977489 | 31020848 |
| [Mus musculus](https://www.ncbi.nlm.nih.gov/Taxonomy/Browser/wwwtax.cgi?mode=Info&id=9606) | [SRR7977490](https://trace.ncbi.nlm.nih.gov/Traces/sra/?run=SRR7474066) | 31020848 |
| [Mus musculus](https://www.ncbi.nlm.nih.gov/Taxonomy/Browser/wwwtax.cgi?mode=Info&id=9606) | [SRR7977482](https://trace.ncbi.nlm.nih.gov/Traces/sra/?run=SRR7474066) | 31020848 |
| [Mus musculus](https://www.ncbi.nlm.nih.gov/Taxonomy/Browser/wwwtax.cgi?mode=Info&id=9606) | [SRR7977483](https://trace.ncbi.nlm.nih.gov/Traces/sra/?run=SRR7474066) | 31020848 |
| [Mus musculus](https://www.ncbi.nlm.nih.gov/Taxonomy/Browser/wwwtax.cgi?mode=Info&id=9606) | [SRR3657469](https://trace.ncbi.nlm.nih.gov/Traces/sra/?run=SRR7474066) | 28358055 |
| [Mus musculus](https://www.ncbi.nlm.nih.gov/Taxonomy/Browser/wwwtax.cgi?mode=Info&id=9606) | [SRR3657470](https://trace.ncbi.nlm.nih.gov/Traces/sra/?run=SRR7474066) | 28358055 |
| [Mus musculus](https://www.ncbi.nlm.nih.gov/Taxonomy/Browser/wwwtax.cgi?mode=Info&id=9606) | [SRR3657471](https://trace.ncbi.nlm.nih.gov/Traces/sra/?run=SRR7474066) | 28358055 |
| [Mus musculus](https://www.ncbi.nlm.nih.gov/Taxonomy/Browser/wwwtax.cgi?mode=Info&id=9606) | [SRR3657472](https://trace.ncbi.nlm.nih.gov/Traces/sra/?run=SRR7474066) | 28358055 |
| [Mus musculus](https://www.ncbi.nlm.nih.gov/Taxonomy/Browser/wwwtax.cgi?mode=Info&id=9606) | [SRR3657473](https://trace.ncbi.nlm.nih.gov/Traces/sra/?run=SRR7474066) | 28358055 |
| [Mus musculus](https://www.ncbi.nlm.nih.gov/Taxonomy/Browser/wwwtax.cgi?mode=Info&id=9606) | [SRR3657474](https://trace.ncbi.nlm.nih.gov/Traces/sra/?run=SRR7474066) | 28358055 |
| [Mus musculus](https://www.ncbi.nlm.nih.gov/Taxonomy/Browser/wwwtax.cgi?mode=Info&id=9606) | [SRR3657475](https://trace.ncbi.nlm.nih.gov/Traces/sra/?run=SRR7474066) | 28358055 |
| [Mus musculus](https://www.ncbi.nlm.nih.gov/Taxonomy/Browser/wwwtax.cgi?mode=Info&id=9606) | [SRR3657476](https://trace.ncbi.nlm.nih.gov/Traces/sra/?run=SRR7474066) | 28358055 |
| [Mus musculus](https://www.ncbi.nlm.nih.gov/Taxonomy/Browser/wwwtax.cgi?mode=Info&id=9606) | [SRR3657477](https://trace.ncbi.nlm.nih.gov/Traces/sra/?run=SRR7474066) | 28358055 |
| [Mus musculus](https://www.ncbi.nlm.nih.gov/Taxonomy/Browser/wwwtax.cgi?mode=Info&id=9606) | [SRR3657478](https://trace.ncbi.nlm.nih.gov/Traces/sra/?run=SRR7474066) | 28358055 |
| Mus musculus | SRR3657479 | 28358055 |
| Mus musculus | SRR3657480 | 28358055 |
| Mus musculus | [SRR13311620](https://trace.ncbi.nlm.nih.gov/Traces/sra/?run=SRR13311620) | 33679870 |
| Mus musculus | SRR13311621 | 33679870 |
| Mus musculus | SRR13311622 | 33679870 |
| Mus musculus | SRR13311623 | 33679870 |
| Mus musculus | SRR13311624 | 33679870 |
| Mus musculus | SRR13311625 | 33679870 |
| [Mus musculus](https://www.ncbi.nlm.nih.gov/Taxonomy/Browser/wwwtax.cgi?mode=Info&id=10090) | [SRR12809340](https://trace.ncbi.nlm.nih.gov/Traces/sra/?run=SRR12809340) | 33996813 |
| Mus musculus | SRR12809341 | 33996813 |
| Mus musculus | SRR12809342 | 33996813 |
| Mus musculus | SRR12809343 | 33996813 |
| Mus musculus | SRR12809344 | 33996813 |
| Mus musculus | SRR12809345 | 33996813 |
| Mus musculus | [SRR12797081](https://trace.ncbi.nlm.nih.gov/Traces/sra/?run=SRR12797081) | 33207694 |
| Mus musculus | SRR12797082 | 33207694 |
| Mus musculus | SRR12797083 | 33207694 |
| Mus musculus | SRR12797084 | 33207694 |
| Mus musculus | SRR12797085 | 33207694 |
| Mus musculus | SRR12797086 | 33207694 |
| Mus musculus | SRR12797087 | 33207694 |
| Mus musculus | SRR12797088 | 33207694 |
| Mus musculus | SRR12797089 | 33207694 |
| Mus musculus | SRR12797090 | 33207694 |
| Mus musculus | SRR12797091 | 33207694 |
| Mus musculus | SRR12797092 | 33207694 |
| Mus musculus | SRR12797093 | 33207694 |
| Mus musculus | SRR12797094 | 33207694 |
| Mus musculus | SRR12797095 | 33207694 |
| Mus musculus | SRR12797096 | 33207694 |
| Mus musculus | SRR12797097 | 33207694 |
| Mus musculus | SRR12797098 | 33207694 |
| [Mus musculus](https://www.ncbi.nlm.nih.gov/Taxonomy/Browser/wwwtax.cgi?mode=Info&id=10090) | SRR12797099 | 33207694 |
| [Mus musculus](https://www.ncbi.nlm.nih.gov/Taxonomy/Browser/wwwtax.cgi?mode=Info&id=10090) | [SRR12797100](https://trace.ncbi.nlm.nih.gov/Traces/sra/?run=SRR6298187) | 33207694 |
| [Mus musculus](https://www.ncbi.nlm.nih.gov/Taxonomy/Browser/wwwtax.cgi?mode=Info&id=10090) | [SRR10004190](https://trace.ncbi.nlm.nih.gov/Traces/sra/?run=SRR6298187) | 31980816 |
| [Mus musculus](https://www.ncbi.nlm.nih.gov/Taxonomy/Browser/wwwtax.cgi?mode=Info&id=10090) | [SRR10004191](https://trace.ncbi.nlm.nih.gov/Traces/sra/?run=SRR6298187) | 31980816 |
| Mus musculus | SRR10004192 | 31980816 |
| Mus musculus | SRR10004193 | 31980816 |
| [Mus musculus](https://www.ncbi.nlm.nih.gov/Taxonomy/Browser/wwwtax.cgi?mode=Info&id=10090) | [SRR8450974](https://trace.ncbi.nlm.nih.gov/Traces/sra/?run=SRR8450974) | 30660194 |
| [Mus musculus](https://www.ncbi.nlm.nih.gov/Taxonomy/Browser/wwwtax.cgi?mode=Info&id=10090) | [SRR7965664](https://trace.ncbi.nlm.nih.gov/Traces/sra/?run=SRR7965664) | 32946063 |
| [Mus musculus](https://www.ncbi.nlm.nih.gov/Taxonomy/Browser/wwwtax.cgi?mode=Info&id=10090) | [SRR7965665](https://trace.ncbi.nlm.nih.gov/Traces/sra/?run=SRR7965664) | 32946063 |
| [Mus musculus](https://www.ncbi.nlm.nih.gov/Taxonomy/Browser/wwwtax.cgi?mode=Info&id=10090) | [SRR6338004](https://trace.ncbi.nlm.nih.gov/Traces/sra/?run=SRR6338004) | / |
| [Mus musculus](https://www.ncbi.nlm.nih.gov/Taxonomy/Browser/wwwtax.cgi?mode=Info&id=10090) | [SRR6338003](https://trace.ncbi.nlm.nih.gov/Traces/sra/?run=SRR6338003) | / |
| Mus musculus | [SRR6298187](https://trace.ncbi.nlm.nih.gov/Traces/sra/?run=SRR5398213) | 29305974 |
| Mus musculus | [SRR6298188](https://trace.ncbi.nlm.nih.gov/Traces/sra/?run=SRR5398213) | 29305974 |
| Mus musculus | [SRR6298189](https://trace.ncbi.nlm.nih.gov/Traces/sra/?run=SRR5398213) | 29305974 |
| Mus musculus | [SRR6298190](https://trace.ncbi.nlm.nih.gov/Traces/sra/?run=SRR5398213) | 29305974 |
| Mus musculus | [SRR5122013](https://trace.ncbi.nlm.nih.gov/Traces/sra/?run=SRR5398213) | 28444238/ 31980816 |
| Mus musculus | SRR5122014 | 28444238/ 31980816 |
| Mus musculus | [SRR5122015](https://trace.ncbi.nlm.nih.gov/Traces/sra/?run=SRR5122011) | 28444238/ 31980816 |
| Mus musculus | [SRR5122016](https://trace.ncbi.nlm.nih.gov/Traces/sra/?run=SRR5122011) | 28444238/ 31980816 |
| Mus musculus | [SRR2983082](https://trace.ncbi.nlm.nih.gov/Traces/sra/?run=SRR2983082) | 2756000 |
| Mus musculus | [SRR2983083](https://trace.ncbi.nlm.nih.gov/Traces/sra/?run=SRR2983082) | 2756000 |
| Oryctolagus cuniculus | [SRR7991980](https://trace.ncbi.nlm.nih.gov/Traces/sra/?run=SRR5122011) | 31130985 |
| Oryctolagus cuniculus | SRR7991981 | 31130985 |
| Oryctolagus cuniculus | [SRR7991982](https://trace.ncbi.nlm.nih.gov/Traces/sra/?run=SRR3479143) | 31130985 |
| Oryctolagus cuniculus | [SRR7991983](https://trace.ncbi.nlm.nih.gov/Traces/sra/?run=SRR3479143) | 31130985 |
| Oryctolagus cuniculus | SRR7991984 | 31130985 |
| Oryctolagus cuniculus | SRR7991985 | 31130985 |
| Oryctolagus cuniculus | SRR7991986 | 31130985 |
| Oryctolagus cuniculus | SRR7991987 | 31130985 |
| Oryctolagus cuniculus | SRR7991979 | 31130985 |
| Rattus norvegicus | SRR9895577 | 31511520 |
| Rattus norvegicus | SRR9895578 | 31511520 |
| Rattus norvegicus | SRR9895579 | 31511520 |
| Rattus norvegicus | SRR16201937 | 34621049 |
| Rattus norvegicus | SRR16201936 | 34621049 |
| Rattus norvegicus | SRR16201935 | 34621049 |
| [Rattus norvegicus](https://www.ncbi.nlm.nih.gov/Taxonomy/Browser/wwwtax.cgi?mode=Info&id=10116) | SRR13194216 | 33323517 |
| Rattus norvegicus | [SRR1555115](https://trace.ncbi.nlm.nih.gov/Traces/sra/?run=SRR1555115) | 26472973 |
| Rattus norvegicus | [SRR1555117](https://trace.ncbi.nlm.nih.gov/Traces/sra/?run=SRR1555117) | 26472973 |
| [Sus scrofa](https://www.ncbi.nlm.nih.gov/Taxonomy/Browser/wwwtax.cgi?mode=Info&id=9823) | [SRR11348425](https://trace.ncbi.nlm.nih.gov/Traces/sra/?run=SRR11348425) | 34781747 |
| Sus scrofa | SRR11348426 | 34781747 |
| Sus scrofa | SRR11348427 | 34781747 |
| Sus scrofa | SRR11348428 | 34781747 |
| Sus scrofa | SRR11348429 | 34781747 |
| Sus scrofa | SRR11348430 | 34781747 |
| zebrafish | SRR9849850 | / |
| zebrafish | SRR9849851 | / |
| zebrafish | [SRR9849852](https://trace.ncbi.nlm.nih.gov/Traces/sra/?run=SRR1049826) | / |
| zebrafish | [SRR9849853](https://trace.ncbi.nlm.nih.gov/Traces/sra/?run=SRR1049826) | / |
| zebrafish | [SRR9849854](https://trace.ncbi.nlm.nih.gov/Traces/sra/?run=SRR1049826) | / |
| zebrafish | [SRR9849855](https://trace.ncbi.nlm.nih.gov/Traces/sra/?run=SRR1049826) | / |
| zebrafish | [SRR9849856](https://trace.ncbi.nlm.nih.gov/Traces/sra/?run=SRR1049826) | / |
| zebrafish | [SRR9849857](https://trace.ncbi.nlm.nih.gov/Traces/sra/?run=SRR1049826) | / |
| zebrafish | [SRR9849858](https://trace.ncbi.nlm.nih.gov/Traces/sra/?run=SRR1049826) | / |
| zebrafish | SRR9849859 | / |
| zebrafish | SRR9849860 | / |
| zebrafish | SRR9849861 | / |
| zebrafish | SRR9849862 | / |
| zebrafish | SRR9849863 | / |
| zebrafish | SRR9849864 | / |
| zebrafish | SRR9849865 | / |
| zebrafish | SRR9849866 | / |

**Table S2.** UCSC genome version and annotation files for each species used in the CircAI database.

| **Species** | **Common name** | **Reference genome** | **URL (fa)** | **URL (gtf)** |
| --- | --- | --- | --- | --- |
| Homo sapiens | Human | hg19 | https://hgdownload.soe.ucsc.edu/goldenPath/hg19/bigZips/hg19.fa.gz | https://hgdownload.soe.ucsc.edu/goldenPath/hg19/bigZips/genes/hg19.ensGene.gtf.gz |
| Mus musculus | Mouse | mm10 | https://hgdownload.soe.ucsc.edu/goldenPath/mm10/bigZips/mm10.fa.gz | https://hgdownload.soe.ucsc.edu/goldenPath/mm10/bigZips/genes/mm10.ensGene.gtf.gz |
| Macaca mulatta | Macaca | rheMac10 | https://hgdownload.soe.ucsc.edu/goldenPath/rheMac10/bigZips/rheMac10.fa.gz | https://hgdownload.soe.ucsc.edu/goldenPath/rheMac10/bigZips/genes/rheMac10.ensGene.gtf.gz |
| Rattus norvegicus | Rat | rn6 | https://hgdownload.soe.ucsc.edu/goldenPath/rn6/bigZips/rn6.fa.gz | https://hgdownload.soe.ucsc.edu/goldenPath/rn6/bigZips/genes/rn6.ensGene.gtf.gz |
| Gallus gallus | Chicken | galGal6 | https://hgdownload.soe.ucsc.edu/goldenPath/galGal6/bigZips/galGal6.fa.gz | https://hgdownload.soe.ucsc.edu/goldenPath/galGal6/bigZips/genes/galGal6.ensGene.gtf.gz |
| Sus scrofa | Pig | susScr11 | https://hgdownload.soe.ucsc.edu/goldenPath/susScr11/bigZips/susScr11.fa.gz | http://hgdownload.soe.ucsc.edu/goldenPath/susScr11/bigZips/genes/susScr11.ensGene.gtf.gz |
| Oryctolagus cuniculus | Rabbit | oryCun2 | https://hgdownload.soe.ucsc.edu/goldenPath/oryCun2/bigZips/oryCun2.fa.gz | https://hgdownload.soe.ucsc.edu/goldenPath/oryCun2/bigZips/genes/oryCun2.ensGene.gtf.gz |
| Danio rerio | Zebrafish | danRer11 | https://hgdownload.soe.ucsc.edu/goldenPath/danRer11/bigZips/danRer11.fa.gz | https://hgdownload.soe.ucsc.edu/goldenPath/danRer11/bigZips/genes/danRer11.ensGene.gtf.gz |
